# Papacy and the Medical Profession in Rome

**Published:** 1869-10

**Authors:** 


### Papacy and the Medical Profession in Rome.

A correspondent of the *Philadelphia Reporter* gives the following in explanation of the low state of medical science in Italy:—

In 1862, during a residence of several weeks in Rome, I spent considerable time in investigating its hospitals and other public charities, as well as the condition of the medical profession and modes of practice in that city, some account of which appeared in the columns of a medical journal published in New York. While its hospitals would do honor to any city, for grandeur and appropriatness of structure and completeness of internal arrangements as well as the kindness and assiduity of the masses, and faithful devotion, the duty of their medical attendants, I found no *esprit du corps* in the medical profession, no medical journals, or medical societies, and indeed very little intercourse among medical men. Medical practice was, probably, about the same as it was one hundred years ago; and, on inquiring further, I found that medical societies were not permitted, or meetings of any professional men, for political reasons; and here I thought I found the cause of the languishing state of medical science in the Papal city. I learn from recent information that, on the 7th May last, the medical and surgical practitioners of Rome received a special "circular" from the "Congregation of Health," inclosing a copy of the dispatch addressed by the Cardinal-Vicar to the President of the Board, styled the "Sacra Consulata." The object of this dispatch is to remind the Board, *by order of the Pope*, of the canons which regulate the administration of the Lord's Supper to the sick, and especially to call attention to the "constitution" laid down by Pius V. bearing the title "Super gregem." In this enactment it is ordered "that no physicians or surgeons shall visit a sick person after the third day of his illness, *unless the invalid shall have called in his confessor, or there exists some reasonable ground for making exception.*" This 'constitution," it is added, was revived by Benedict XIII., at the council held at Rome in the year 1725, with the additions under Clause XXXII of the "Pœnit." And "in case the physician or surgeon continue his visits after the third day, the sick man not having made his confession in the interim, he shall be visited with the penalty of the major excommunication, or "Latac Sententiæ," exclusively vested in the Sovereign Pontiff and local bishops, and *may likewise be punished with very severe penalties.*"

I am uncertain whether it is the patient or the physician who is to be punished, but, as I understand it, it is the latter. I may, however, be mistaken.

Such are the rigid proscriptions with which the Cardinal-Vicar requires the medical practitioners in Rome minutely to conform. The Papal press alleges that this revival of an obsolete injunction shows a pious concern for the salvation of souls; but surely it is a great infraction of individual liberty, constituting the medical attendant into an inquisitor, as regards his patient's conscience, and depriving the latter of medical aid should he neglect or decline to confess to a priest! It is this domineering, inquisitorial spirit which characterizes Papal sacerdotalism in Rome, which interferes with the progress of science there, and the enlightened spirit of the age, and which especially depresses and discourages the medical practitioners of that city. L.

